# Association Between the Use of Psychotropic Medications and the Risk of COVID-19 Infection Among Long-term Inpatients With Serious Mental Illness in a New York State–wide Psychiatric Hospital System

**DOI:** 10.1001/jamanetworkopen.2022.10743

**Published:** 2022-05-06

**Authors:** Katlyn Nemani, Sharifa Z. Williams, Mark Olfson, Emily Leckman-Westin, Molly Finnerty, Jammie Kammer, Thomas E. Smith, Daniel J. Silverman, Jean-Pierre Lindenmayer, Gillian Capichioni, James Clelland, Donald C. Goff

**Affiliations:** 1 Nathan S. Kline Institute for Psychiatric Research, Orangeburg, New York; 2 Department of Psychiatry, New York University Langone Medical Center, New York; 3 Department of Psychiatry, Columbia University Irving Medical Center, New York, New York; 4 New York State Office of Mental Health, New York

## Abstract

**Importance:**

Individuals with serious mental illness are at increased risk of severe COVID-19 infection. Several psychotropic medications have been identified as potential therapeutic agents to prevent or treat COVID-19 but have not been systematically examined in this population.

**Objective:**

To evaluate the associations between the use of psychotropic medications and the risk of COVID-19 infection among adults with serious mental illness receiving long-term inpatient psychiatric treatment.

**Design, Setting, and Participants:**

This retrospective cohort study assessed adults with serious mental illness hospitalized in a statewide psychiatric hospital system in New York between March 8 and July 1, 2020. The final date of follow-up was December 1, 2020. The study included 1958 consecutive adult inpatients with serious mental illness (affective or nonaffective psychoses) who received testing for SARS-CoV-2 by reverse transcriptase–polymerase chain reaction or antinucleocapsid antibodies and were continuously hospitalized from March 8 until medical discharge or July 1, 2020.

**Exposures:**

Psychotropic medications prescribed prior to COVID-19 testing.

**Main Outcomes and Measures:**

COVID-19 infection was the primary outcome, defined by a positive SARS-CoV-2 reverse transcriptase–polymerase chain reaction or antibody test result. The secondary outcome was COVID-19–related death among patients with laboratory-confirmed infection.

**Results:**

Of the 2087 adult inpatients with serious mental illness continuously hospitalized during the study period, 1958 (93.8%) underwent testing and were included in the study; 1442 (73.6%) were men, and the mean (SD) age was 51.4 (14.3) years. A total of 969 patients (49.5%) had laboratory-confirmed COVID-19 infection that occurred while they were hospitalized; of those, 38 (3.9%) died. The use of second-generation antipsychotic medications, as a class, was associated with decreased odds of infection (odds ratio [OR], 0.62; 95% CI, 0.45-0.86), whereas the use of mood stabilizers was associated with increased odds of infection (OR, 1.23; 95% CI, 1.03-1.47). In a multivariable model of individual medications, the use of paliperidone was associated with decreased odds of infection (OR, 0.59; 95% CI, 0.41-0.84), and the use of valproic acid was associated with increased odds of infection (OR, 1.39; 95% CI, 1.10-1.76). Clozapine use was associated with reduced odds of mortality in unadjusted analyses (unadjusted OR, 0.25; 95% CI, 0.10-0.62; fully adjusted OR, 0.43; 95% CI, 0.17-1.12).

**Conclusions and Relevance:**

In this cohort study of adults hospitalized with serious mental illness, the use of second-generation antipsychotic medications was associated with decreased risk of COVID-19 infection, whereas the use of valproic acid was associated with increased risk. Further research is needed to assess the mechanisms that underlie these findings.

## Key Points

**Question:**

Is psychotropic medication use associated with differences in the risk of COVID-19 infection among adults with serious mental illness?

**Findings:**

In this cohort study of 1958 inpatients with serious mental illness in a statewide psychiatric hospital system, the use of second-generation antipsychotic medications was associated with a decreased risk of COVID-19 infection; the largest association was observed with the use of paliperidone. Valproic acid use was associated with an increased risk of infection.

**Meaning:**

These results suggest that individual psychotropic medications are associated with differential risks of COVID-19 infection among patients with serious mental illness.

## Teaser

This cohort study evaluates the associations between the use of psychotropic medications and the risk of COVID-19 infection among adults with serious mental illness receiving long-term inpatient psychiatric treatment.

## Introduction

Individuals with serious mental illness are especially vulnerable to COVID-19. Patients with psychiatric disorders are more likely to have medical comorbidities associated with worse outcomes and have a higher mortality rate from COVID-19 independent of these medical risk factors.^1,2,3,4,5^ Among psychiatric diagnoses, schizophrenia is associated with the greatest increase in mortality risk.^1,3,5,6^ Although increased mortality risk after COVID-19 infection has been consistently observed among patients with psychiatric disorders, some studies have found lower rates of COVID-19 infection among patients with major psychiatric disorders.^3,6,7^ This finding may reflect a true decrease in infection rates, possibly owing to social isolation, or failure to detect infection among individuals who do not receive testing.^8^ In contrast, inpatients residing in psychiatric treatment facilities are at high risk of viral exposure and often have greater access to testing.

Identifying factors associated with the risk of infection among inpatients with serious mental illness is of critical importance given their susceptibility to severe infection. A study of adults with serious mental illness infected with COVID-19 found equivalent mortality rates among those taking antipsychotic medications compared with those not taking antipsychotic medications,^9^ suggesting that antipsychotic treatment is unlikely to account for increased mortality risk. However, individual medications within and across pharmacologic classes may differ in their associations with infection and adverse outcomes. Several psychotropic medications—including some first-generation antipsychotics (haloperidol^10^ and chlorpromazine^11^) and antidepressants (fluvoxamine, in particular^12^) were identified as potential therapeutic agents based on in vitro evidence of anti–SARS-CoV-2 activity. Clinical evidence to support these in vitro findings has been mixed. Although several studies have established an association between the use of antidepressants^13,14,15^ and a less severe course of infection in patients with COVID-19, small observational studies have found no association between the use of haloperidol^16^ or chlorpromazine^17^ and the severity of COVID-19 infection. These medications and several other psychotropic drugs, including second-generation antipsychotics, may affect the host response to COVID-19 by modifying the balance between proinflammatory and anti-inflammatory cytokines and through other mechanisms.^18,19^ Several of these medications are common treatments for adults with serious mental illness, but their associations with the risk of COVID-19 infection have not been systematically examined in this population, to our knowledge.

The primary aim of this study was to assess the risk of COVID-19 infection associated with psychopharmacologic treatments among adult long-term inpatients with serious mental illness in a statewide psychiatric hospital system in New York. We also aimed to assess the risk of COVID-19 mortality among patients with laboratory-confirmed infection.

## Methods

### Study Design and Participants

In this retrospective cohort study, we used data from the electronic health record (EHR) and a centralized COVID-19 registry used by all psychiatric hospitals operated by the New York State (NYS) Office of Mental Health (OMH). The study protocol was reviewed by the institutional review board of the Nathan S. Kline Institute for Psychiatric Research and deemed exempt from the requirement of informed consent based on use of deidentified data and designation of non–human participants research. This study followed the Strengthening the Reporting of Observational Studies in Epidemiology (STROBE) reporting guideline for cohort studies.

The NYS-OMH operates psychiatric hospitals across the state that provide treatment for adults with serious mental illness in civil units as well as specially designated forensic centers. Adults admitted to civil units meet criteria for involuntary commitment under the Mental Hygiene Law and are typically referred from inpatient psychiatric units in community hospitals for treatment of severe and persistent psychiatric symptoms. In contrast to the relatively brief length of stay at acute care hospitals, the mean length of stay in NYS-OMH facilities varies from 6 months to several years. All hospitals have nursing staff coverage 24 hours a day, 7 days a week, and ensure medication adherence through direct observation while patients are taking prescribed medication.

At the beginning of the COVID-19 pandemic in March 2020, the NYS-OMH implemented a centralized COVID-19 registry that included COVID-19 test results and clinical status for every patient in a NYS-OMH facility, including medical transfers to other facilities for COVID-19 treatment and COVID-19–related deaths. The registry was updated daily, with required reporting retroactive to March 1, 2020. SARS-CoV-2 reverse transcriptase–polymerase chain reaction (RT-PCR) testing of suspected cases began March 1, 2020, using multiple commercial laboratories; as of March 24, 2020, RT-PCR testing was performed by a single laboratory (Bioreference Laboratories Inc). The NYS-OMH made universal testing available to all hospitalized patients beginning May 7, 2020; patients were offered screening by RT-PCR and antibody testing using the ARCHITECT SARS CoV-2 antinucleocapsid protein immunoglobulin G (IgG) assay (Abbott). This assay has a reported sensitivity of 100% and specificity of 99.6% for detecting SARS-CoV-2 within several days after infection,^20^ with detectable antibodies for at least 3 to 6 months after infection.^21^ As of July 1, 2020, 93% of patients in a NYS-OMH facility had received COVID-19 testing.

Included in this study were adult inpatients (≥18 years of age) with serious mental illness (schizophrenia, schizoaffective disorder, bipolar I disorder, or depression with psychotic features) who received testing for SARS-CoV-2 by RT-PCR or by serum IgG assay and were continuously hospitalized from March 8, 2020, until medical discharge for COVID-19 or July 1, 2020. Patients who were admitted within 30 days prior to testing were excluded because we assessed medication exposure during the 30 days prior to testing, and information about medications could not be confirmed for patients admitted during this assessment window (eFigure in the Supplement). The primary outcome was infection; patients with any positive test result were considered positive. The secondary outcome was COVID-19–related deaths among patients with laboratory-confirmed infection, as reported in the registry and confirmed in the EHR. Deaths were monitored through December 1, 2020.

### Procedures

The exposure of interest was psychotropic medications taken prior to COVID-19 testing. Medication data were extracted from the EHR. Patients were considered exposed to a medication if it was (1) prescribed prior to their index test date, (2) used for at least 7 days, and (3) received via scheduled administration (as-needed medications were excluded). Medications were grouped by pharmacologic class: first-generation antipsychotic, second-generation antipsychotic, mood stabilizer, benzodiazepine, or antidepressant (eTable 1 in the Supplement).

The medication exposure period was defined based on the date of SARS-CoV-2 test and the type of test (RT-PCR or antibody). The date of the first positive test result was used as the index date for patients with any positive test result. A positive RT-PCR test result was considered a proxy of active infection, and the exposure period was limited to 30 days. Patients who were transferred to a medical facility for COVID-19 treatment prior to receiving confirmatory testing were also considered to have active infection, and the exposure period included 30 days prior to transfer. For patients without a positive RT-PCR test result or medical transfer for COVID-19 treatment, the date of the SARS-CoV-2 IgG test was used as the index date. Because this was considered a measure of prior infection, the exposure period included the full study period prior to testing (starting from March 8, 2020).

The following covariates were extracted from the EHR and assessed as potential confounders based on their known or hypothesized association with medication exposure and COVID-19 infection and/or death: age, sex, race and ethnicity, body mass index, smoking status, medical comorbid conditions, and psychiatric diagnoses. Race and ethnicity were based on patient self-report and categorized as Asian or Pacific Islander, Black, Latinx, White, and other (including Native American, multiple races, or “other race”) or unknown. Missing data for race and ethnicity and smoking status were assigned to unknown categories. Medical comorbid conditions included chronic respiratory disease, diabetes, heart disease, and hypertension, using *International Statistical Classification of Diseases and Related Health Problems, Tenth Revision* medical billing category codes (eTable 2 in the Supplement). Psychiatric diagnoses were determined based on admission diagnosis and grouped into nonaffective psychotic disorders (schizophrenia and delusional disorder) and affective psychotic disorders (schizoaffective disorder, bipolar I disorder, and major depressive disorder with psychotic features). Hospital size (number of inpatients) as well as geographic location were also included as model covariates.

### Statistical Analysis

We first used unadjusted binary logistic regression to estimate associations between baseline characteristics and psychopharmacologic classes, with COVID-19 infection as the primary outcome (Table 1) and COVID-19–related death (Table 2) as the secondary outcome.

**Table 1.** Baseline Characteristics of All Psychiatric Inpatients and COVID-19–Positive Patients

Characteristic	All patients, No. (%) (N = 1958)	Patients with COVID-19^a^	
		No. (%) (n = 969 [49.5%])	Crude OR (95% CI)
Clinical characteristics			
Age, mean (SD), y	51.4 (14.3)	52.8 (14.2)	1.01 (1.01-1.02)^b^
Age group, y			
18-44	634 (32.4)	269 (27.8)	1 [Reference]
45-54	371 (19.0)	186 (19.2)	1.36 (1.06-1.77)^b^
55-64	570 (29.1)	295 (30.4)	1.46 (1.16-1.83)^b^
≥65	383 (19.6)	219 (22.6)	1.81 (1.40-2.34)^b^
Sex			
Male	1442 (73.6)	723 (74.6)	1 [Reference]
Female	516 (26.4)	246 (25.4)	0.91 (0.74-1.11)
Race and ethnicity			
Asian or Pacific Islander	83 (4.2)	54 (5.6)	2.43 (1.51-3.90)^b^
Black	761 (38.9)	410 (42.3)	1.53 (1.25-1.87)^b^
Latinx	303 (15.5)	156 (16.1)	1.39 (1.06-1.81)^b^
White	747 (38.2)	324 (33.4)	1 [Reference]
Other or unknown^c^	64 (3.3)	25 (2.6)	0.84 (0.50-1.41)
Smoking status			
Nonsmoker	1021 (52.1)	492 (50.8)	1 [Reference]
Smoker	419 (21.4)	172 (17.8)	0.75 (0.60-0.94)^b^
Status not available	518 (26.5)	305 (31.5)	1.54 (1.24-1.91)^b^
BMI group			
Normal (≤25)	530 (27.1)	283 (29.2)	1 [Reference]
Overweight (26-30)	735 (37.5)	372 (38.4)	0.89 (0.72-1.12)
Low-risk obesity (31-35)	457 (23.3)	206 (21.3)	0.72 (0.56-0.92)^b^
Moderate- to high-risk obesity (>35)	236 (12.1)	108 (11.1)	0.74 (0.54-1.00)^b^
Chronic respiratory disease			
Yes	136 (6.9)	80 (8.3)	1.50 (1.05-2.14)^b^
No	1822 (93.1)	889 (91.7)	1 [Reference]
Diabetes			
Yes	240 (12.3)	136 (14.0)	1.39 (1.06-1.82)^b^
No	1718 (87.7)	833 (86.0)	1 [Reference]
Heart disease			
Yes	70 (3.6)	47 (4.9)	2.14 (1.29-3.55)^b^
No	1888 (96.4)	922 (95.1)	1 [Reference]
Hypertension			
Yes	368 (18.8)	214 (22.1)	1.54 (1.22-1.93)^b^
No	1590 (81.2)	755 (77.9)	1 [Reference]
Affective psychotic disorder			
Yes^d^	911 (46.5)	452 (46.7)	1.01 (0.85-1.21)
No^e^	1047 (53.5)	517 (53.4)	1 [Reference]
Facility size, mean (SD) No. of patients	166.8 (74.3)	199.8 (60.9)	1.01 (1.01-1.02)^b^
Hospital region			
New York City and Long Island	1099 (56.1)	653 (67.4)	1 [Reference]
Hudson River	508 (25.9)	307 (31.7)	1.04 (0.84-1.29)
Central or Western New York	351 (17.9)	9 (0.9)	0.02 (0.01-0.04)^b^
Psychotropic medication class			
First-generation antipsychotic			
Yes	1198 (61.2)	611 (63.1)	1.17 (0.97-1.40)^b^
No	760 (38.8)	358 (36.9)	1 [Reference]
Second-generation antipsychotic			
Yes	1794 (91.6)	870 (89.8)	0.62 (0.45-0.86)^b^
No	164 (8.4)	99 (10.2)	1 [Reference]
Mood stabilizers			
Yes	1043 (53.3)	541 (55.8)	1.23 (1.03-1.47)^b^
No	915 (46.7)	428 (44.2)	1 [Reference]
Benzodiazepines			
Yes	1016 (51.9)	502 (51.8)	0.99 (0.83-1.19)
No	942 (48.1)	467 (48.2)	1 [Reference]
Antidepressants			
Yes	547 (27.9)	261 (26.9)	0.91 (0.74-1.10)
No	1411 (72.1)	708 (73.1)	1 [Reference]

Abbreviations: BMI, body mass index (calculated as weight in kilograms divided by height in meters squared); OR, odds ratio.

^a^ Estimates show the crude associations between clinical characteristics, psychotropic medication class, and COVID-19 infection from binary logistic regression.

^b^ *P* < .10.

^c^ Includes Native American, multiple races, or “other race.”

^d^ Includes schizoaffective disorder, bipolar I disorder, and major depressive disorder with psychotic features.

^e^ Includes schizophrenia, delusional disorder, and schizotypal disorder.

**Table 2.** Baseline Characteristics of All COVID-19–Positive Psychiatric Inpatients and COVID-19–Related Deaths

Characteristic	All patients with COVID-19, No. (%) (N = 969)	COVID-19–related deaths^a^		
		No. (%) (n = 38 [3.9%])	Crude OR (95% CI)
Clinical characteristics			
Age, mean (SD), y	52.8 (14.2)	67.0 (10.6)	1.12 (1.08-1.16)^b^
Age group, y			
18-44	269 (27.8)	1 (2.6)	1 [Reference]
45-54	186 (19.2)	2 (5.3)	2.91 (0.26-32.36)
55-64	295 (30.4)	9 (23.7)	8.43 (1.06-67.00)^b^
≥65	219 (22.6)	26 (68.4)	36.10 (4.86-268.28)^b^
Sex			
Male	723 (74.6)	29 (76.3)	1 [Reference]
Female	246 (25.4)	9 (23.7)	0.91 (0.42-1.95)
Race and ethnicity			
Asian or Pacific Islander	54 (5.6)	5 (13.2)	2.44 (0.83-7.15)
Black	410 (42.3)	13 (34.2)	0.78 (0.36-1.71)
Latinx	156 (16.1)	5 (13.2)	0.79 (0.28-2.26)
White	324 (33.4)	13 (34.2)	1 [Reference]
Other or unknown^c^	25 (2.6)	2 (5.3)	2.08 (0.44-9.78)
Smoking status			
Nonsmoker	492 (50.8)	15 (39.5)	1 [Reference]
Smoker	172 (17.8)	6 (15.8)	1.15 (0.44-3.01)
Status not available	305 (31.5)	17 (44.7)	1.88 (0.92-3.82)^b^
BMI group			
Normal (≤25)	283 (29.2)	11 (28.9)	1 [Reference]
Overweight (26-30)	372 (38.4)	16 (42.1)	1.11 (0.51-2.43)
Low-risk obesity (31-35)	206 (21.3)	6 (15.8)	0.74 (0.27-2.04)
Moderate- to high-risk obesity (>35)	108 (11.1)	5 (13.2)	1.20 (0.41-3.54)
Chronic respiratory disease			
Yes	80 (8.3)	4 (10.5)	1.32 (0.46-3.83)
No	889 (91.7)	34 (89.5)	1 [Reference]
Diabetes			
Yes	136 (14.0)	12 (31.6)	3.00 (1.48-6.11)^b^
No	833 (86.0)	26 (68.4)	1 [Reference]
Heart disease			
Yes	47 (4.9)	8 (21.1)	6.09 (2.63-14.17)^b^
No	922 (95.1)	30 (78.9)	1 [Reference]
Hypertension			
Yes	214 (22.1)	17 (44.7)	3.02 (1.56-5.83)^b^
No	755 (77.9)	21 (55.3)	1 [Reference]
Affective psychotic disorder			
Yes^d^	452 (46.6)	16 (42.1)	0.83 (0.43-1.59)
No^e^	517 (53.4)	22 (57.9)	1 [Reference]
Facility size, mean (SD) No. of patients	199.8 (60.9)	213.8 (54.2)	1.00 (1.00-1.01)
Hospital region			
New York City and Long Island	653 (67.4)	23 (60.5)	1 [Reference]
Hudson River	307 (31.7)	15 (39.5)	1.41 (0.72-2.74)
Central or Western New York	9 (0.9)	0	NA
Psychotropic medication class			
First-generation antipsychotic			
Yes	611 (63.0)	23 (60.5)	0.89 (0.46-1.74)
No	358 (37.0)	15 (39.5)	1 [Reference]
Second-generation antipsychotic			
Yes	870 (89.8)	33 (86.8)	0.74 (0.28-1.94)
No	99 (10.2)	5 (13.2)	1 [Reference]
Mood stabilizers			
Yes	541 (55.8)	19 (50.0)	0.78 (0.41-1.50)
No	428 (44.2)	19 (50.0)	1 [Reference]
Benzodiazepines			
Yes	502 (51.8)	16 (42.1)	0.67 (0.35,1.28)
No	467 (48.2)	22 (57.9)	1 [Reference]
Antidepressants			
Yes	261 (26.9)	6 (15.8)	0.50 (0.21-1.20)^b^
No	708 (73.1)	32 (84.2)	1 [Reference]

Abbreviations: BMI, body mass index (calculated as weight in kilograms divided by height in meters squared); NA, not applicable; OR, odds ratio.

^a^ Estimates show the crude associations between clinical characteristics, psychotropic medication class, and COVID-19 death among inpatients with COVID-19.

^b^ *P* < .10.

^c^ Includes Native American, multiple races, or “other race.”

^d^ Includes schizoaffective disorder, bipolar I disorder, and major depressive disorder with psychotic features.

^e^ Includes schizophrenia, delusional disorder, and schizotypal disorder.

Statistical tests were 2-sided. Multivariable statistical modeling retained individual medications with at least 5% exposure in the study population from psychopharmacologic classes identified as being associated with infection at the *P* < .10 significance level.^22^ Binary logistic regression was used to estimate the association between use of each medication and infection using odds ratios (ORs) and 95% CIs. Three types of ORs were estimated: (1) unadjusted, (2) adjusted for age and sex, and (3) fully adjusted for age and sex in addition to covariates that were significantly associated with infection in binary logistic regression (*P* < .05). Because patients could have been exposed to multiple medications within and across drug classes, medications were retained in each step to empirically adjust for polypharmacy. A Bonferroni-type correction was used to adjust for multiple comparisons within medication classes.

A similar approach was used to assess the association between medication exposure and mortality among patients with a positive SARS-CoV-2 test result. Medications retained for statistical modeling included those from drug classes associated with mortality (*P* < .10) and medications significantly associated with infection (*P* < .05) after adjusting for age and sex. All analyses were conducted using SAS, version 9.4 (SAS Institute Inc).

## Results

A total of 2087 adults with serious mental illness were continuously inpatients at 18 NYS-OMH psychiatric hospitals between March 8 and July 1, 2020; of these 2087 inpatients, 125 (6.0%) were excluded owing to lack of testing information, and 4 were excluded owing to admission within 30 days prior to the test result (eFigure in the Supplement). The eligible cohort included 1958 patients with a mean (SD) age of 51.4 (4.3) years; 1442 (73.6%) were male and 516 (26.4%) were female (Table 1). Of these 1958 patients, 969 (49.5%) had laboratory-confirmed COVID-19 infection; 572 cases (59.0%) were identified by antibody testing. Among the 969 inpatients with confirmed infections, 38 (3.9%) were determined to have had a COVID-19–related death (Table 2).

Patients prescribed second-generation antipsychotics were less likely to test positive for COVID-19 infection than those not prescribed second-generation antipsychotics (OR, 0.62; 95% CI, 0.45-0.86), whereas patients prescribed mood stabilizers were more likely to test positive than patients who were not exposed to medications in this class (OR, 1.23; 95% CI, 1.03-1.47) (Table 1). First-generation antipsychotic use was associated with a nonsignificant increase in odds of infection (OR, 1.17; 95% CI, 0.97-1.40; *P* = .08). No difference in the rate of infection was observed in association with benzodiazepine or antidepressant exposure.

Each of the second-generation antipsychotics (olanzapine, clozapine, risperidone, aripiprazole, quetiapine, and paliperidone), mood stabilizers (valproic acid, lithium, and lamotrigine), and first-generation antipsychotics (haloperidol, fluphenazine, and chlorpromazine) with at least 5% exposure in the study population were retained for regression modeling of infection (Table 3). After adjustment for age and sex, decreased odds of infection were observed in association with use of chlorpromazine (OR, 0.59; 95% CI, 0.40-0.86), clozapine (OR, 0.79; 95% CI, 0.64-0.98), paliperidone (OR, 0.59; 95% CI, 0.42-0.81), risperidone (OR, 0.67; 95% CI, 0.53-0.86), and olanzapine (OR, 0.70; 95% CI, 0.58-0.86). Conversely, valproic acid use was associated with increased odds of infection after adjusting for age and sex (OR, 1.36; 95% CI, 1.12-1.65). After full adjustment for sociodemographic characteristics (including age, sex, race and ethnicity, facility size, and region), medical risk factors (including body mass index, chronic respiratory disease, diabetes, hypertension, and heart disease), and exposure to other medications, use of paliperidone remained significantly associated with decreased infection (OR, 0.59; 95% CI, 0.41-0.84), whereas use of valproic acid remained significantly associated with increased infection (OR, 1.39; 95% CI, 1.10-1.76) (Figure). Results of a sensitivity analysis limited to RT-PCR testing, with a 30-day medication exposure period for all participants, can be found in eTable 3 in the Supplement.

**Table 3.** Incidence and Adjusted Odds Ratios of COVID-19 Infection by Psychotropic Medication Exposures

Psychotropic medication	All patients, No. (N = 1958)	Patients with COVID-19, No. (%) (n = 969 [49.5%])	Odds ratio (95% CI)		
			Medications only	Adjusted for age and sex	Fully adjusted^a^
First-generation antipsychotics					
Haloperidol	886	453 (51.1)	1.08 (0.90-1.31)	1.14 (0.94-1.38)	0.86 (0.69-1.08)
Fluphenazine	274	138 (50.4)	1.01 (0.77-1.32)	1.04 (0.79-1.37)	0.88 (0.64-1.21)
Chlorpromazine	134	47 (35.1)	0.55 (0.38-0.81)^b^	0.59 (0.40-0.86)^b^	0.99 (0.63-1.54)
Second-generation antipsychotics					
Olanzapine	860	398 (46.3)	0.69 (0.57-0.84)^c^	0.70 (0.58-0.86)^c^	0.82 (0.65-1.04)
Clozapine	712	341 (47.9)	0.73 (0.59-0.90)^c^	0.79 (0.64-0.98)	0.99 (0.77-1.27)
Risperidone	377	157 (41.6)	0.66 (0.52-0.84)^c^	0.67 (0.53-0.86)^c^	0.75 (0.57-1.00)
Aripiprazole	266	131 (49.2)	0.97 (0.74-1.27)	1.03 (0.79-1.35)	0.97 (0.72-1.33)
Quetiapine	262	126 (48.1)	0.84 (0.64-1.10)	0.85 (0.64-1.11)	0.72 (0.52-1.00)
Paliperidone	186	67 (36.0)	0.57 (0.41-0.79)^c^	0.59 (0.42-0.81)^c^	0.59 (0.41-0.84)^c^
Mood stabilizers					
Valproic acid	688	363 (52.8)	1.34 (1.10-1.63)^b^	1.36 (1.12-1.65)^b^	1.39 (1.10-1.76)^b^
Lithium	375	190 (50.7)	1.14 (0.90-1.44)	1.17 (0.92-1.48)	0.95 (0.72-1.24)
Lamotrigine	136	74 (54.4)	1.35 (0.94-1.94)	1.36 (0.94-1.94)	1.40 (0.91-2.16)

^a^ The fully adjusted odds ratios controlled for age, sex, race and ethnicity, facility size, hospital region, body mass index, chronic respiratory disease, diabetes, hypertension, heart disease, and exposure to medications in the model.

^b^ *P* < .02, where α′ = .05/3 = .017 to correct for multiple comparisons.

^c^ *P* < .001, where α′ = .05/6 = .0008 to correct for multiple comparisons.

**Figure.**
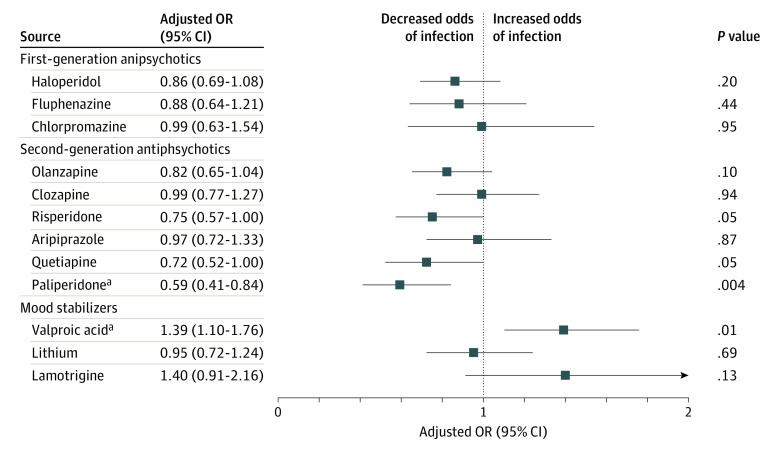
Adjusted Odds Ratios (ORs) for Infection by Psychotropic Medication Exposure The fully adjusted ORs controlled for age, sex, race and ethnicity, hospital region, body mass index, chronic respiratory disease, diabetes, hypertension, heart disease, and exposure to medications in the model. ^a^Significant associations at the Bonferroni-adjusted level of significance.

In the secondary analysis of mortality of 969 inpatients with infection, antidepressant medications were retained for statistical modeling based on bivariate analysis of medication class (OR, 0.50; 95% CI, 0.21-1.20; *P* = .10). Chlorpromazine, clozapine, paliperidone, risperidone, olanzapine, and valproic acid were retained based on their significant association with infection after adjusting for age and sex. Clozapine was the only medication associated with decreased odds of mortality in the unadjusted model (OR, 0.25, 95% 0.10-0.62); this association did not remain statistically significant after adjusting for age and sex (OR, 0.40; 95% CI, 0.16-1.03) or after additional adjustment for medical risk factors (OR, 0.43; 95% CI, 0.17-1.12) (Table 4). There were no deaths among COVID-19–positive patients prescribed chlorpromazine (n = 53) or escitalopram (n = 48). A list of individual psychotropic medications prescribed to the full study cohort and by outcomes (infection and mortality) is included in eTable 1 in the Supplement.

**Table 4.** Case Fatality and Adjusted Odds Ratios of COVID-19 Mortality by Psychotropic Medication Exposures

Psychotropic medication	Patients with COVID, No. (n = 969)	COVID-19–related deaths, No. (%) (n = 38 [3.9%])	Odds ratio (95% CI)		
			Medications only	Adjusted for age and sex	Fully adjusted^a^
First-generation antipsychotic					
Chlorpromazine^b^	47	0	NA	NA	NA
Second-generation antipsychotic					
Olanzapine	398	14 (3.5)	0.54 (0.26-1.09)	0.61 (0.30-1.27)	0.61 (0.29-1.29)
Clozapine	341	6 (1.8)	0.25 (0.10-0.62)^c^	0.40 (0.16-1.03)	0.43 (0.17-1.12)
Risperidone	157	3 (1.9)	0.32 (0.10-1.07)	0.35 (0.10-1.20)	0.42 (0.12-1.45)
Paliperidone	67	2 (3.0)	0.62 (0.14-2.71)	0.51 (0.11-2.32)	0.54 (0.12-2.49)
Mood stabilizers					
Valproic acid	363	16 (4.4)	1.33 (0.68-2.59)	1.58 (0.78-3.16)	1.48 (0.72-3.03)
Antidepressants					
Sertraline	62	1 (1.6)	0.36 (0.05-2.67)	0.46 (0.06-3.52)	0.53 (0.07-4.08)
Citalopram	70	1 (1.4)	0.36 (0.05-2.67)	0.38 (0.05-2.94)	0.46 (0.06-3.57)
Escitalopram^b^	47	0	NA	NA	NA

Abbreviation: NA, not applicable.

^a^ The fully adjusted odds ratios controlled for age, sex, diabetes, hypertension, heart disease, and other medications in the model.

^b^ Could not be retained for statistical modeling owing to 0 deaths.

^c^ *P* < .01, where α′ = .05/4 = .0125 to correct for multiple comparisons.

## Discussion

We investigated COVID-19 infection and mortality among long-term inpatients with serious mental illness in a NYS-operated psychiatric hospital system during the first wave of the pandemic, with a focus on the association between exposures to psychotropic medications and adverse outcomes. Nearly half of the patients in this cohort had laboratory-confirmed COVID-19 infection, and infection-related fatality was more than 4 times higher than estimates from the general population in New York during the same time period.^23^ This finding is consistent with prior studies that have found increased rates of infection in congregate settings^24,25^ and increased mortality after infection among patients with serious mental illness.^1,5,6,7^ The potential harms and benefits associated with several psychotropic medications have been explored in preclinical and clinical studies since the start of the COVID-19 pandemic,^19^ but, to our knowledge, this is the largest study to systematically assess associations between the use of individual medications and the risk of COVID-19 infection among inpatients with serious mental illness.

The use of second-generation antipsychotics, as a class, was associated with decreased infection. The largest effect size was observed in association with paliperidone. The use of second-generation antipsychotics was also found to have a protective association with mortality, although the association was not statistically significant, likely owing to insufficient sample size. These findings are contrary to what would be expected based on in vitro evidence showing that first-generation antipsychotics (specifically, haloperidol), but not second-generation antipsychotics, interact with sigma-1-receptors to reduce SARS-CoV-2 replication.^10^ However, there are other drug-protein interactions that may interfere with the viral life cycle. SARS-CoV-2 3c-like protease and viral RNA-dependent polymerase are critical enzymes for viral protein processing and genome replication, respectively. An in silico screening analysis identified paliperidone as one of the leading drugs to bind these enzymes with high affinity.^26^

Although there have been concerns about clozapine use during the pandemic as a risk factor for pneumonia and potential toxic effects during acute infection,^27^ clozapine use was not associated with an increased risk of COVID-19 infection or death in the present study. In fact, unadjusted estimates suggested a significant protective association. This finding stands in contrast to prior EHR studies that found an increased risk of COVID-19 infection associated with clozapine treatment^28,29^ but is consistent with a prior study limited to inpatients that found a lower risk of infection and a lower risk of symptomatic disease in association with clozapine use.^30^ One potential explanation for this discrepancy is surveillance bias because patients receiving clozapine in the general community may be more likely than other outpatients to undergo testing for COVID-19. Because 94% of patients in this cohort underwent testing, surveillance bias was less likely. Furthermore, this is the largest study to date of COVID-19–infected patients prescribed clozapine. Clozapine is unique among antipsychotics in its ability to enhance the T helper cell type 1 response that supports antiviral immune response and is blunted in schizophrenia.^31^ Further research is needed to determine whether clozapine may protect against severe COVID-19 infection.

To our knowledge, this is the first study to assess the association between valproic acid use and COVID-19 risk. Although it has been hypothesized that the use of valproic acid could reduce the risk of COVID-19 infection or reduce viral load by its association with angiotensin-converting enzyme 2 and transmembrane serine protease 2,^32^ we found a significant increase in infection and a similar (although nonsignificant) increase in mortality. Valproic acid downregulates angiotensin-converting enzyme 2 in endothelial cells,^33^ and several studies have shown that the downregulation of membrane-bound angiotensin-converting enzyme 2 may impair immune function and contribute to poor outcomes in the setting of COVID-19 infection.^34^ Additional factors may confound the association between valproic acid use and COVID-19 risk. For example, patients are typically prescribed mood stabilizers, such as valproic acid, to manage mood lability and other manic symptoms; these symptoms may negatively affect adherence to infection prevention measures, such as mask wearing and social distancing.

Risk of mortality was lower among patients taking antidepressants. Although the association was not statistically significant in this cohort, this finding is consistent with larger studies that found reduced risk of adverse outcomes associated with antidepressant use.^13,14,15,35^ There were no COVID-19–related deaths among patients prescribed escitalopram (n = 88), venlafaxine (n = 53), bupropion (n = 43), or fluvoxamine (n = 25).

### Strengths and Limitations

This study has several strengths. Restricting the sample to inpatients with chronic psychotic disorders minimized differences in risk associated with community exposures, underlying psychiatric diagnosis, medication adherence, and access to care, thus facilitating assessment of the association between psychotropic medications and outcomes. Implementation of universal screening and use of antibody testing to detect prior infection helped identify patients who may not have received confirmatory testing by RT-PCR when infected owing to lack of test availability or other factors.

This study also has several limitations. During the study period, the pandemic was at its first peak in New York. Interventions, including regular testing of staff and a statewide vaccination campaign, which successfully limited the spread of COVID-19 during the second surge of the virus,^36^ had not yet been implemented. The availability of tests and criteria for testing evolved during the study period and varied across facilities. Duration of medication use, medication dose, and polypharmacy may have been associated with differences in outcomes and were not explored in this analysis. In addition, medication exposure after infection could not be evaluated for patients who were transferred to outside facilities for medical treatment. Medication adherence, although likely high, was not directly assessed. Additionally, the study population represents a subset of adults with serious mental illness: those with persistent and severe illness, chronically exposed to psychotropic medications, who reside in long-term state psychiatric hospitals. The association between the use of psychotropic medications and the risk of COVID-19 infection may differ between patients with less severe psychiatric disease and those without prior medication exposure.

## Conclusions

Exposures to several psychotropic medications were associated with risk of COVID-19 infection among inpatients with serious mental illness; decreased risk was observed with the use of second-generation antipsychotics, with paliperidone use associated with the largest effect size. Valproic acid use was associated with an increased risk of infection. Further research is needed to replicate our findings, evaluate their generalizability, and explore underlying mechanisms.

## Notes

Supplement. **eFigure.** Study Profile **eTable 1.** Psychotropic Medications Exposure in Full Cohort and by Infection and Death **eTable 2.** *ICD-9/10* Category Codes Used to Identify Medical Conditions and Psychiatric Diagnoses **eTable 3.** Incidence and Adjusted Odds Ratios of COVID-19 Infection by Psychotropic Medication Exposure in Patients with PCR Testing
