# Supplementary material for: Association Between the Use of Psychotropic Medications and the Risk of COVID-19 Infection Among Long-term Inpatients With Serious Mental Illness in a New York State–wide Psychiatric Hospital System
Source: JAMA Netw Open. 2022 May 6;5(5):e2210743. doi: 10.1001/jamanetworkopen.2022.10743 (PMC9077485; doi:10.1001/jamanetworkopen.2022.10743)
Supplement: Supplement. — eFigure. Study Profile eTable 1. Psychotropic Medications Exposure in Full Cohort and by Infection and Death eTable 2. ICD-9/10 Category Codes Used to Identify Medical Conditions and Psychiatric Diagnoses eTable 3. Incidence and Adjusted Odds Ratios of COVID-19 Infection by Psychotropic Medication Exposure in Patients with PCR Testing [file jamanetwopen-e2210743-s001.pdf]

Supplementary Online Content

Nemani K, Williams SZ, Olfson M, et al. Association between the use of psychotropic medications and the risk of COVID-19 infection among long-term inpatients with serious mental illness in a New York State–wide psychiatric hospital system. *JAMA Netw Open.* 2022;5(5):e2210743. doi:10.1001/jamanetworkopen.2022.10743

eFigure. Study Profile
eTable 1. Psychotropic Medications Exposure in Full Cohort and by Infection and Death
eTable 2. *ICD-9/10* Category Codes Used to Identify Medical Conditions and Psychiatric Diagnoses
eTable 3. Incidence and Adjusted Odds Ratios of COVID-19 Infection by Psychotropic Medication Exposure in Patients with PCR Testing

This supplementary material has been provided by the authors to give readers additional information about their work.

© 2022 Nemani K et al. *JAMA Network Open.*

eFigure. Study Profile

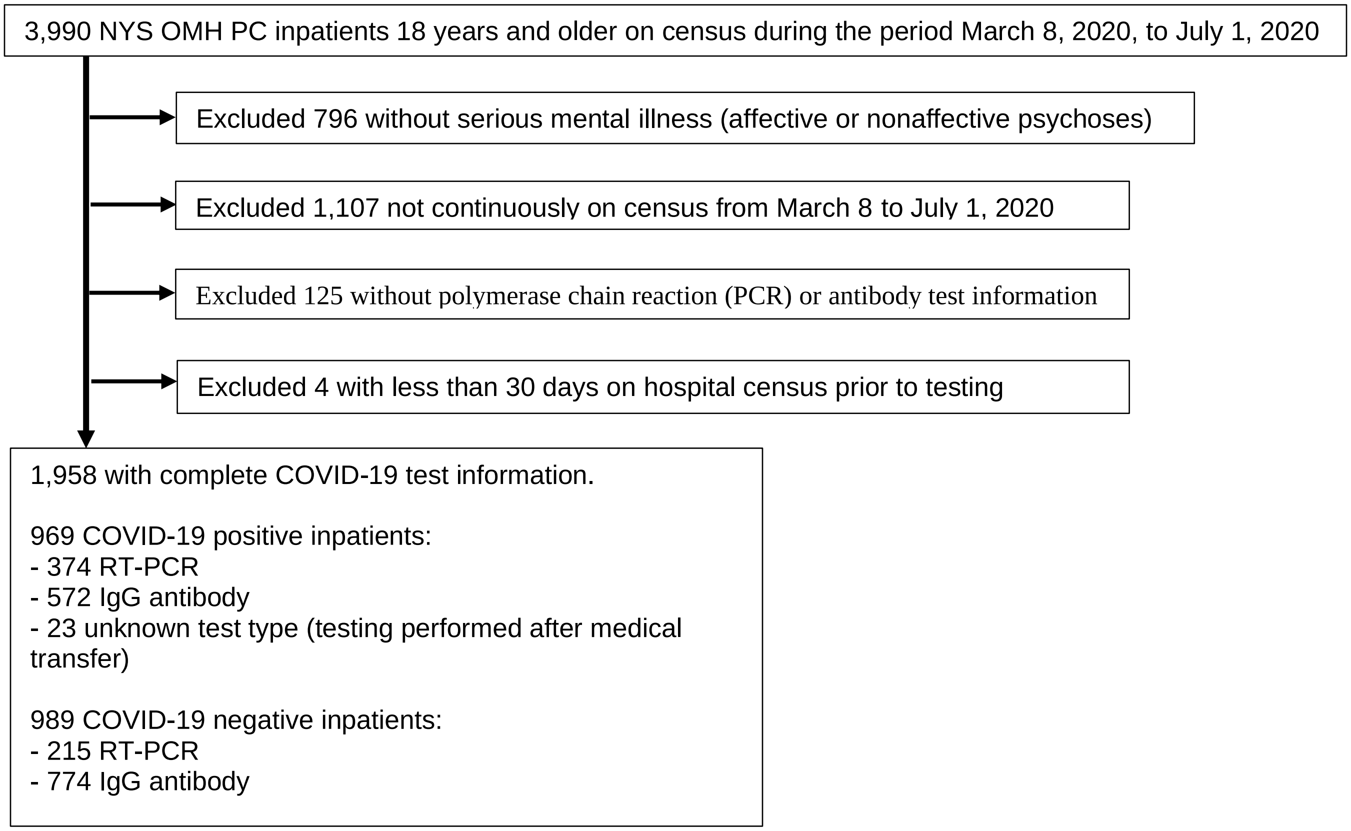

© 2022 Nemani K et al. *JAMA Network Open.*

eTable 1. Psychotropic Medications Exposure in Full Cohort and by Infection and Death[a]

Psychotropic medication	Total, n (%)	COVID-19 positive, n (%)	COVID-19 death, n (%)
All	1,958 (100·0)	969 (100.0)	38 (100.0)
**First-generation antipsychotics**	1,198 (61·2)	611 (63.1)	23 (60.5)
Haloperidol	886 (45·2)	453 (46.8)	19 (50.0)
Fluphenazine	274 (14·0)	138 (14.2)	3 (7.9)
Chlorpromazine	134 (6·8)	47 (4.9)	0 (0·0)
Perphenazine	37 (1·9)	20 (2.1)	0 (0·0)
Loxapine	31 (1·6)	19 (2.0)	0 (0·0)
Prochlorperazine	10 (0·5)	3 (0.3)	0 (0·0)
Thioridazine	5 (0·3)	4 (0.4)	0 (0·0)
Pimozide	1 (0·1)	1 (0.1)	1 (2.6)
Trifluoperazine	1 (0·1)	0 (0·0)	0 (0·0)
**Second-generation antipsychotics**	1,794 (91·6)	870 (89.8)	33 (86.8)
Olanzapine	860 (43.9)	398 (41.1)	14 (36·8)
Clozapine	712 (36·4)	341 (35.2)	6 (15·8)
Risperidone	377 (19·2)	157 (16.2)	3 (7·9)
Aripiprazole	266 (13·6)	131 (13.5)	3 (7.9)
Quetiapine	262 (13·4)	126 (13.0)	6 (15.8)
Paliperidone	186 (9·5)	67 (6.9)	2 (5.3)
Ziprasidone	56 (2·9)	19 (2.0)	0 (0·0)
Lurasidone	11 (0·6)	3 (0·3)	0 (0·0)
Cariprazine	5 (0·3)	1 (0.1)	0 (0·0)
Pimavanserin	1 (0·1)	0 (0·0)	0 (0·0)
**Mood stabilizers**	1,043 (53·3)	541 (55·8)	19 (50.0)
Valproic acid	688 (35·1)	363 (37.5)	16 (42·1)
Lithium	375 (19·2)	190 (19.6)	6 (15.8)
Lamotrigine	136 (7·0)	74 (7.6)	2 (5.3)
Topiramate	38 (1·9)	20 (2.1)	0 (0·0)
**Antidepressants**	547 (27·9)	261 (26.9)	6 (15.8)
Sertraline	145 (7·4)	62 (6.4)	1 (2·6)
Citalopram	134 (6·8)	70 (7.2)	1 (2.6)
Escitalopram	88 (4·5)	47 (4.9)	0 (0·0)
Mirtazapine	82 (4·2)	35 (3.6)	3 (7.9)
Fluoxetine	66 (3·4)	29 (3.0)	0 (0·0)
Venlafaxine	53 (2·7)	26 (2.7)	0 (0·0)
Bupropion	43 (2·2)	16 (1.7)	0 (0·0)
Paroxetine	29 (1·5)	22 (2.3)	1 (2·6)
Fluvoxamine	25 (1·3)	16 (1.7)	0 (0·0)
Duloxetine	18 (0·9)	5 (0.5)	0 (0·0)
Clomipramine	13 (0·7)	6 (0.6)	0 (0·0)
Doxepin	7 (0·4)	0 (0·0)	0 (0·0)
Amitriptyline	3 (0·2)	0 (0·0)	0 (0·0)
Imipramine	3 (0·2)	2 (0.2)	0 (0·0)
Nortriptyline	2 (0·1)	1 (0.1)	0 (0·0)
**Benzodiazepines**	1,016 (51·9)	502 (51.8)	16 (42·1)
Lorazepam	810 (41·4)	404 (41.7)	12 (31·6)
Clonazepam	420 (21·4)	198 (20.4)	4 (10.5)
Diazepam	19 (1·0)	11 (1.1)	0 (0·0)
Alprazolam	1 (0·1)	1 (0.1)	0 (0·0)
Temazepam	1 (0·1)	1 (0..1)	0 (0·0)

[a]Patients can be prescribed multiple medications both within and across psychopharmacologic classes.

© 2022 Nemani K et al. *JAMA Network Open.*

eTable 2. *ICD-9/10* Category Codes Used to Identify Medical Conditions and Psychiatric Diagnoses

**Condition**	***ICD-9/10* category code**
Bipolar disorder	F30* or F31*
Chronic respiratory disease	J43-J45
Delusional disorder	F22
Diabetes	E08-11, E13
Heart disease	I21-I25, I30-I52
Hypertension	I10, I11, I15, I16
Major depressive disorder with psychotic features	F2.3 or F33.3
Schizoaffective disorder	F25*
Schizophrenia	F20*

© 2022 Nemani K et al. *JAMA Network Open.*

eTable 3. Incidence and Adjusted Odds Ratios of COVID-19 Infection by Psychotropic Medication Exposure in Patients with PCR Testing

Psychotropic medication	COVID-19 positive n (%)	Medications only	Age-Sex Adjusted	Fully Adjusted[a]
		OR (95% CI)	OR (95% CI)	OR (95% CI)
All (n=1,363)	374 (27.4)	--	--	--
First generation antipsychotic				
Haloperidol (n=610)	177 (29.0)	1.04 (0.81, 1.35)	1.24 (0.87, 1.46)	0.84 (0.62, 1.13)
Fluphenazine (n=187)	51 (27.3)	0.93 (0.64,1.35)	0.96 (0.66,1.39)	0.81 (0.53,1.22)
Chlorpromazine (n=101)	14 (13.9)	0.42 (0.23,0.75)	0.45 (0.24,0.82)	0.71 (0.37,1.37)
Second generation antipsychotic				
Olanzapine (n=618)	156 (25.2)	0.67 (0.52,0.88)	0.70 (0.54,0.92)	0.85 (0.62,1.16)
Clozapine (n=493)	122 (24.8)	0.62 (0.47,0.82)	0.73 (0.55,0.98)	0.99 (0.70,1.39)
Risperidone (n=282)	62 (22.0)	0.68 (0.48,0.94)	0.69 (0.50,0.97)	0.78 (0.53,1.13)
Aripiprazole (n=174)	39 (22.4)	0.68 (0.46,1.01)	0.77 (0.52,1.14)	0.67 (0.43,1.04)
Quetiapine (n=185)	49 (26.5)	0.84 (0.58,1.21)	0.85 (0.59,1.23)	0.75 (0.49,1.15)
Paliperidone (n=144)	25 (17.4)	0.51 (0.32,0.81)	0.54 (0.34,0.87)	0.55 (0.33,0.92)
Mood stabilizers				
Valproic acid (n=467)	142 (30.4)	1.40 (1.08,1.82)	1.41 (1.09,1.83)	1.57 (1.15,2.13)
Lithium (n=261)	76 (29.1)	1.22 (0.90,1.67)	1.28 (0.93,1.75)	1.10 (0.77,1.58)
Lamotrigine (n=99)	37 (37.4)	1.88 (1.21,2.92)	1.83 (1.17,2.87)	2.05 (1.20,3.49)

[a]The fully adjusted odds ratios control for age, sex, race/ethnicity, facility size, hospital region, BMI, chronic respiratory disease, diabetes, hypertension, heart disease, and exposure to medications in the model.

© 2022 Nemani K et al. *JAMA Network Open.*
